# Native aortic valve endocarditis with *Morganella morganii* in a patient with multiple myeloma and valvular amyloidosis: a case report

**DOI:** 10.1186/s12879-019-4511-4

**Published:** 2019-11-10

**Authors:** Renée van Bentum, Judith Nieken, Esther de Waal, Mels Hoogendoorn

**Affiliations:** 1 Department of Internal Medicine, Medical Center Leeuwarden, Henri Dunantweg 2, 8934 AD Leeuwarden, The Netherlands; 2 Pathologie Friesland, Center for Pathology, Jelsumerstraat 6a, 8917 EN Leeuwarden, The Netherlands

**Keywords:** Multiple myeloma, Infective endocarditis, *Morganella morganii*, Amyloidosis

## Abstract

**Background:**

Patients with multiple myeloma (MM) are known to be immune incompetent and experience higher incidences of infectious diseases. However, infective endocarditis (IE) is rarely observed in patients with MM and *Morganella morganii (M. morganii*) has rarely been associated with IE.

**Case presentation:**

A 72-year-old female receiving 4th line treatment for MM presented with fever and concomitant confusion. Urinary culture revealed growth of *Escherichia coli*, wherefore broadspectrum penicillin and high-dose corticosteroids were initiated. However, blood cultures showed growth of *M. morganii*. Fluoroquinolone was added due to penicillin-resistance of the *Morganella* species. Two days after admission, the patient acutely deteriorated with hemodynamic instability. Gentamicin and high dose corticosteroids were added. Echocardiography showed marked aortic valve vegetation with severe aortic valve regurgitation, leading to the diagnosis of bacterial endocarditis of the native aortic valve. Shortly after diagnosis, the patient died. At autopsy, vegetation with gram-negative rods in the native aortic valve was observed, confirming the diagnosis of *M. morganii*-endocarditis. Additional staining for amyloid confirmed advanced light-chain (AL) amyloidosis with extensive amyloid depositions of the aortic valve and valvular damage as complications of her MM.

**Conclusions:**

Our case suggests that IE with *M. morganii* was facilitated by the combination of the cardiac amyloidosis with valvular impairment and the profound immune deficiency caused by the several chemo-immunomodulatory treatment lines and the MM itself. This case further illustrates that awareness for rare opportunistic infections in an era with growing potential of combined chemoimmunotherapy is warranted.

## Background

Patients with multiple myeloma (MM) are known to be immune incompetent and experience higher incidence of infectious diseases [1, 2]. However, compared to other infections, infective endocarditis (IE) is relatively rare for patients with MM. Here, we describe a patient, suffering from MM, with a rapidly fatal *M. morganii* endocarditis. Since *M. morganii* has only seldom been associated with IE [3, 4] and IE is rarely diagnosed in patients with MM [2], we hypothesize that in this patient the MM may have played a pivotal role in initiating IE.

## Case presentation

A 72-year-old Dutch female presented to the emergency department with fever and concomitant confusion. Three years before this admission, a symptomatic MM was diagnosed for which melphalan, prednisolone and bortezomib were initiated. Eighteen months before presentation lenalidomide in combination with dexamethasone was given for relapse of her MM, and 2 months later cyclophosphamide was added to this regimen. Six months later she progressed and received third line therapy consisting of bortezomib, thalidomide and dexamethasone. Four months before this presentation the MM was regarded refractory to bortezomib and immunomodulatory drugs, thus immunotherapy with weekly daratumumab in combination with prednisone was offered. Although amyloidosis was suspected due to proteinuria, no confirmation by fat tissue biopsy was performed. One year before presentation an echocardiography showed no structural abnormalities of valves or heart. At admission, no (septic) shock was present with vital signs showing mild tachycardia (100 beats per minute), blood pressure of 110/60 mmHg, temperature of 37.2 °C and respiratory rate of 15 per minute. History revealed fever, pollakisuria and confusion since 2 days. At physical examination no cardiac murmur, no signs of systemic major emboli, normal pulmonary auscultation and no signs for an acute abdomen or heart failure were found. No infusaport was in situ. Laboratory investigations showed mildly elevated leukocyte count (10.9 × 10^9^/L) together with elevated C-reactive protein (127 mg/L). Kidney function, electrolytes and liver enzymes were normal. Immunoglobulin gamma was 31.4 g/L, which was stable over time and hypo-albuminaemia was also stable at 11 g/L. Electrocardiogram was unchanged when compared to 1 year earlier, without signs of arrhythmia, AV-blocks or myocardial ischemia. Pulmonary X-ray was normal. Urine tests were compatible with a urinary tract infection with positive nitrite and leukocytosis. Dipstick screening showed also signs of albuminuria and microscopic hematuria, which were not investigated further at this point. Intravenous amoxicillin with clavulanic acid (1000/200 mg 4 times daily) was initiated corresponding to Dutch guidelines for urinary tract infections. Hydrocortisone (100 mg bolus followed by continuous infusion of 100 mg per 24 h) was initiated because of suspected tertiary adrenal insufficiency due to prolonged use of prednisolone. One day after admission the results of urine culture confirmed bacterial growth of *Escherichia coli*. In contrast, all four blood cultures showed growth of *M. morganii*. Ciprofloxacin (200 mg 2 times daily) was added due to the penicillinresistance of the *Morganella* species. There was no evident portal of entry for the *M. morganii,* though no specific work-up was done.

**Fig. 1:**
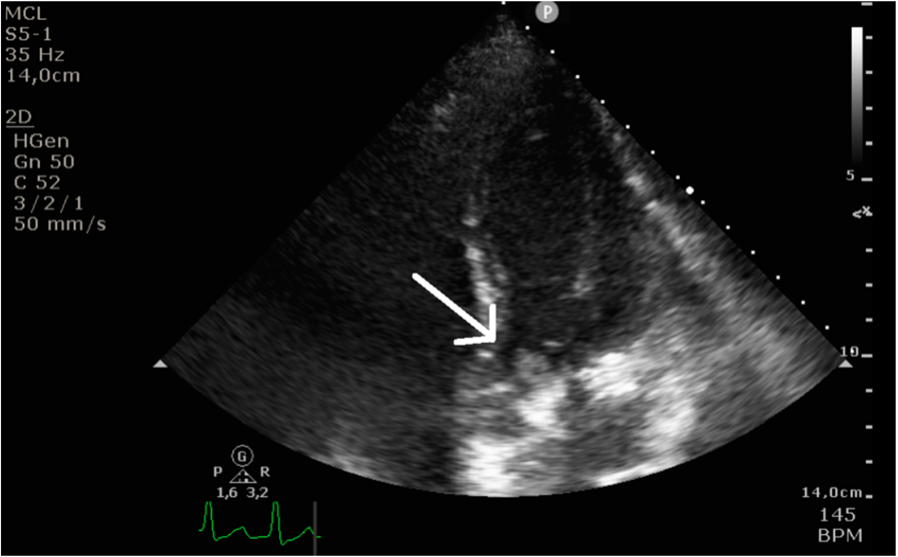
Transthoracic echocardiography showing a mobile structure adhered to the native aortic valve (white arrow)

Two days after admission, the patient acutely deteriorated with hemodynamic instability. Under the diagnosis of septic shock gentamicin (4 mg per kg) and once more hydrocortisone (100 mg bolus injection) were administered without improvement of hemodynamic status after 1 h. Transthoracic echocardiography showed severe aortic regurgitation and a mobile structure, 10 by 10 mm, attached to left or non-coronary cusp, strongly suspective of vegetation (Fig. 1). These findings led to the diagnosis of bacterial endocarditis of the native aortic valve. Half an hour after diagnosis, the patient died due to ongoing hypoperfusion and refractory shock.

**Fig. 2:**
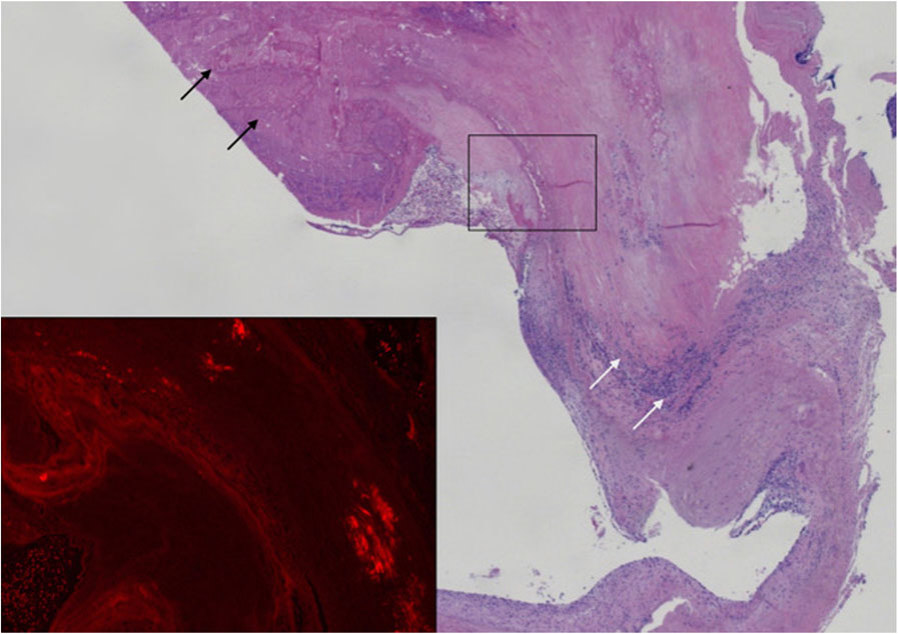
Overview of the aortic valve with inflammation (white arrows) and adherent vegetation (black arrows) (H&E, 25 x). Inset: amyloid deposition confirmed by Congo red fluorescence microscopy, showing diagnostic bright orange-red areas (100 x)

At autopsy, vegetation with gram-negative rods in the native aortic valve was observed, consistent with a diagnosis of *M. morganii*-endocarditis. Additional staining for amyloid confirmed advanced light-chain (AL) amyloidosis in heart, kidneys and spleen. The aortic valve itself contained patchy amyloid depositions near the adherent vegetation (Fig. 2). These observations led to our hypothesis that the patient became susceptible to IE caused by the opportunistic pathogen *M. morganii* due to valvular endothelial damage related to amyloid depositions and a severely compromised immune system.

## Discussion and conclusions

We here present a rare case of a patient with MM and concomitant AL amyloidosis, who died of a *M. morganii* IE. Several features of this case report we consider as unique.

First, IE is seldom diagnosed in MM patients, although these patients are known to be more susceptible for infections [1, 2]. In the early phase after diagnosis a marked humoral immunodeficiency is present, due to impaired lymphocyte function, plasma cell dysfunction and hypogammaglobulinemia. In later phases immunodeficiency can deteriorate due to hospitalization, chemotherapy-induced neutropenia and immunomodulatory therapies. The latter may become increasingly important since new combined chemo-immunotherapy seem to contribute to increased susceptibility for infections [1]. Overall, a 7-fold increased risk of infections is present in MM patients [2]. Furthermore, almost half of the early deaths and 22% of total deaths of patients with MM are infection-related [2, 5]. Pneumonia and bacteraemia are most commonly reported [1, 2, 5]. The most common pathogens are the encapsulated bacteria *Streptococcus pneumoniae, Staphylococcus aureus* and *Escherichia coli* [1, 2, 5]. Despite the susceptibility for infections and the common occurrence of bacteraemia, the reported incidence of IE in MM patients remains relatively low (although still higher than the general Dutch population (0.37% vs 0,006%)) [2, 6]. Our patient received four lines of therapy and might have been prone to the rare *M. morganii* opportunistic infection. Although literature describing infections in MM-patients receiving new combined chemo-immunotherapy regimens is scarce, it can be hypothesized that incidences of serious opportunistic infections may increase in patients receiving high dosages of corticosteroids, high dose chemotherapy and immunomodulatory agents. Furthermore, with the introduction of the new treatment modalities, survival of MM patients has significantly improved and thus more infectious complications can be encountered.

Second, *M. morganii* has rarely been associated with IE and to the best of our knowledge this is the first case of a fatal *M. morganii* IE in an immune compromised patient [3, 4]. IE predominantly occurs in patients with valvular abnormalities such as stenotic or regurgent valves, valve prostheses or recent surgery. Causative organisms in most cases are gram-positive cocci. *M. morganii* is a gramnegative rod that is naturally present in the gastrointestinal tract and rarely causes disease in healthy subjects [7]. The urinary tract, skin and the hepatobiliary tract are the most common sites when infection does occur. Bacteraemia has been described, mostly in immune compromised patients [8, 9]. Although rare, *M. morganii* bacteraemia has a high mortality [7]. In addition, intrinsic resistance for β-lactam antibiotics could possibly lead to delay of adequate empiric treatment, which further underscores the importance of this micro-organism. Our patient might have been susceptible to IE caused by this opportunistic pathogen due to the MM and the profound immune deficiency caused by the several treatment lines given.

Third, autopsy confirmed both the presence of systemic and cardiac light-chain amyloidosis and an infective aortic endocarditis near patchy amyloid deposits within the native aortic valve. Therefore, we hypothesize that amyloidosis of the aortic valve might have enhanced the susceptibility of the patient to IE. Amyloidosis is a heterogenic group of rare diseases, sharing the key finding of extracellular depositions of insoluble fibrillar proteins in organs and tissue such as kidney, liver and heart. In patients with MM, systemic amyloidosis can develop from the accumulation of light-chain amyloid (AL amyloid) depositions. Cardiac amyloidosis is a restrictive cardiomyopathy due to fibril deposition in the myocardium in both ventricles, causing heart failure of both sides. Mainly rightsided heart failure can be severe and is sometimes the presenting symptom of cardiac amyloidosis [10]. Cardiac amyloidosis is the most serious form of systemic amyloidosis and is responsible for 40% of deaths in patients with systemic amyloidosis [11]. Involvement of heart valves is present in 13% of cases according to Cacoub et al. [12]. *M. morganii* is known to be capable of infecting the urinary tract in the presence of urinary catheters, due to hemagglutinin that enhances adherence to the catheter. Whether hemagglutinin might also enhance the adherence to amyloid depositions of the heart valves is speculative [13]. Since echocardiography 1 year prior to admission showed a structurally normal heart without signs of cardiac amyloidosis, we speculate that her AL systemic amyloidosis occurred late in her disease course and was rapidly progressive in the last year. In our patient no diagnostic fat tissue biopsy was performed due to limited therapeutic consequences. No association between cardiac amyloidosis and IE was found in literature. However, in a review of 34 IE cases a patient was described with the combination of cardiac amyloidosis, valvular amyloid depositions and IE, similar to our patient [14].

In conclusion, our case showed that *M. morganii* is indeed capable of causing IE*.* We hypothesize that this rare opportunistic infection in our patient was facilitated by the combination of the cardiac amyloidosis with valvular impairment and the profound immune deficiency caused by the several chemo-immunomodulatory treatment lines and the MM itself. This case further illustrates that awareness for rare opportunistic infections in an era with growing potential of combined chemoimmunotherapy is warranted.

## Data Availability

Not applicable.

## Abbreviations

IE: Infective endocarditis
M. morganii: Morganella morganii
MM: Multiple myeloma
